# Amino Acid Intakes Are Inversely Associated with Arterial Stiffness and Central Blood Pressure in Women^1^,^2^

**DOI:** 10.3945/jn.115.214700

**Published:** 2015-07-22

**Authors:** Amy Jennings, Alex MacGregor, Ailsa Welch, Phil Chowienczyk, Tim Spector, Aedín Cassidy

**Affiliations:** 3 Department of Nutrition, Norwich Medical School, University of East Anglia, Norwich, United Kingdom; and; 4 Department of Twin Research and Genetic Epidemiology, King’s College London, London, United Kingdom

**Keywords:** protein, amino acids, blood pressure, arterial stiffness, cardiovascular

## Abstract

**Background:** Although data suggest that intakes of total protein and specific amino acids (AAs) reduce blood pressure, data on other cardiovascular disease risk factors are limited.

**Objective:** We examined associations between intakes of AAs with known mechanistic links to cardiovascular health and direct measures of arterial stiffness, central blood pressure, and atherosclerosis.

**Methods:** In a cross-sectional study of 1898 female twins aged 18–75 y from the TwinsUK registry, intakes of 7 cardioprotective AAs (arginine, cysteine, glutamic acid, glycine, histidine, leucine, and tyrosine) were calculated from food-frequency questionnaires. Direct measures of arterial stiffness and atherosclerosis included central systolic blood pressure (cSBP), mean arterial pressure (MAP), augmentation index (AI), pulse wave velocity (PWV), and intima–media thickness (IMT). ANCOVA was used to assess the associations between endpoints of arterial stiffness and intake (per quintile), adjusting for potential confounders.

**Results:** In multivariable analyses, higher intakes of total protein and 7 potentially cardioprotective AAs were associated with lower cSBP, MAP, and PWV. Higher intakes of glutamic acid, leucine, and tyrosine were most strongly associated with PWV, with respective differences of −0.4 ± 0.2 m/s (*P*-trend = 0.02), −0.4 ± 0.2 m/s (*P*-trend = 0.03), and −0.4 ± 0.2 m/s (*P*-trend = 0.03), comparing extreme quintiles. There was a significant interaction between AA intakes and protein source, and higher intakes of AAs from vegetable sources were associated with lower central blood pressure and AI. Higher intakes of glutamic acid, leucine, and tyrosine from animal sources were associated with lower PWV.

**Conclusions:** These data provide evidence to suggest that intakes of several AAs are associated with cardiovascular benefits beyond blood pressure reduction in healthy women. The magnitude of the observed associations was similar to those previously reported for other lifestyle factors. Increasing intakes of these AAs could be an important and readily achievable way to reduce cardiovascular disease risk.

## Introduction

Dietary protein intake has received increased attention for its role in preventing cardiovascular disease, especially in relation to blood pressure–lowering effects. A higher total protein intake has been inversely associated with both systolic and diastolic blood pressure in a number of observational studies (1). Furthermore, a meta-analysis of 40 randomized, controlled trials reported significant decreases in blood pressure with increased intakes of total (systolic −1.8 mm Hg and diastolic −1.2 mm Hg), animal (systolic −2.5 mm Hg and diastolic −1.0 mm Hg), and vegetable (systolic −2.3 mm Hg and diastolic −1.3 mm Hg) protein (2). Other studies, however, have found that it is protein from vegetable sources that is inversely related to blood pressure, with no significant effects for animal protein (3, 4). Furthermore, plant protein has been shown to be a strong marker of dietary quality, whereas the findings for animal protein are currently equivocal (5).

The mechanisms explaining the vasoactive properties of dietary protein are likely to be dependent on the amino acid composition and the source of the protein. A number of amino acids, including glutamic acid, arginine, glycine, cysteine, and histidine, have been shown to modulate concentrations of nitric oxide, a potent vasodilator (6–9). In addition, leucine has been shown to modulate insulin/phosphoinositide 3-kinase signaling in animal models (10), and cysteine has been shown to increase glucose uptake and concentrations of glucose transporter 3 and glucose transporter 4 in vitro; in rat models, dietary cysteine has been shown to reduce insulin resistance and glucose intolerance (11, 12). Tyrosine is thought to reduce blood pressure by stimulating catecholamine synthesis in the brain (13), whereas histidine has been shown to inhibit vascular expression of the angiotensin converting enzyme mRNA in hypertensive rats (9). To our knowledge, only one previous cross-sectional study has examined associations between intakes of amino acids with established vasoprotective properties and blood pressure and showed that a higher intake of tyrosine was related to a 2.4 mm Hg lower systolic blood pressure (14). Other cross-sectional and longitudinal studies, which have examined associations between blood pressure and all amino acids, reported inverse associations between higher intakes of glutamic acid, histidine, and tyrosine and systolic blood pressure (15–17). Higher intakes of glutamic acid and cysteine has also been associated with a decreased risk of stroke mortality and incidence in women (18, 19).

There is evidence to suggest that measures of central arterial function are better predictors of cardiovascular events than brachial blood pressure (20). Arterial stiffness provides assessment of both the structure and function of the artery, and pulse wave velocity (PWV)^5^ is considered to be the gold standard measurement and most consistently predictive of cardiovascular disease risk (20). The measurement of central blood pressure with the use of the augmentation index (AI) is considered to be a strong predictor of atherosclerosis (20), and intima–media thickness (IMT) predicts future incidence of coronary heart disease (21).

To our knowledge, no previous cross-sectional studies have examined associations between the intakes of these 7 potentially cardioprotective amino acids and in vivo measures of arterial stiffness and atherosclerosis. Therefore, we examined associations between these 7 amino acids, which have known mechanistic links to blood pressure, and endpoints of arterial stiffness (PWV and AI), atherosclerosis (IMT), and central and peripheral blood pressure in a cohort of healthy women aged 18–75y. On the basis of previous research, it was hypothesized that participants with higher intakes of arginine, cysteine, glutamic acid, glycine, histidine, leucine, and tyrosine would have improved arterial stiffness and central blood pressure.

## Methods

### 

#### Study population.

The current study used data collected from female twin pairs in the TwinsUK registry, a nationwide registry that consists of adult twin volunteers recruited from the general population through national media campaigns in the United Kingdom (22). All participants were unaware of the specific hypotheses being tested, and were not selected on the basis of the variables being studied. Zygosity was derived by questionnaire and confirmed by multiplex DNA fingerprinting (PE Applied Biosystems). Informed consent was obtained from all participants and ethical approval for the study was gained from St. Thomas’s Hospital Research Ethics committee. The participants included in this analysis were female twin pairs aged 18–75 y, and were a sample of the total population group (*n* = 5725) who had completed both FFQs and had a clinical assessment of arterial stiffness and atherosclerosis progression between 1996 and 2000. Of the 5119 twins who completed an FFQ, 36% (*n* = 1857) were excluded for having an incomplete FFQ (>10 food items were left blank) or implausible energy intake [the ratio of the FFQ-derived estimate of total energy intake to estimated basal metabolic rate fell 2 SDs outside the mean ratio (<0.52 or >2.58) for this cohort] (23). A further 27% of participants (*n* = 1364) did not attend a clinical session for vascular assessment, which left 1898 participants (949 twin pairs) for the current analysis. PWV and IMT were measured in a subset of 900 participants of the total population who were part of a planned longitudinal study on the heritability of arterial stiffness, of which 728 (81%) completed a valid FFQ and were included in these analyses. A power calculation related to a clinically significant association with a PWV of 0.4 m/s at an α level of 0.05 revealed 98% power to see an association with 728 participants. This population has previously been shown to be representative of the general population in terms of blood pressure and dietary intake (22, 23).

#### Assessment of arterial stiffness, central blood pressure and IMT.

Measurements were performed during a single visit to a quiet, temperature-controlled (22–24°C) clinical laboratory. Peripheral systolic blood pressure (pSBP) and peripheral diastolic blood pressure (pDBP) were measured by a trained nurse who used an automated cuff sphygmomanometer (OMRON HEM713C) with the participant in the seated position for at least 3 min before taking 3 measurements. Measures of central blood pressure and AI were obtained with the subject in a supine position with the use of the SphygmoCor system (Atcor Medical), as described previously for this cohort (24). Intraoperator and interoperator reproducibility, expressed as intraclass correlations, were 0.82 and 0.84, respectively (25). In a subset of the population (*n* = 728) who were part of a study on the heritability of arterial stiffness, PWV was calculated from sequential recordings of electrocardiogram-referenced carotid and femoral pressure waveforms obtained by tonometry with the use of the same device and transducer, as previous described (26).

#### Assessment of amino acid intake.

Participants completed a 131-item validated FFQ (27, 28). Intakes of amino acids were derived predominantly with the use of UK food composition data but with additional data from the USDA (29, 30). The intake of glutamic acid in these datasets relates to glutamic acid plus glutamine. Values for 18 individual amino acids were assigned to each of the foods listed in the FFQ, and for recipes, values for each ingredient in the dishes were assigned with the use of the data sources described above. When values for total protein from the amino acid database and the latest UK food composition tables differed, the amino acid composition of the food items was modified to match the most up-to-date data (29). Intake of individual amino acids was calculated as the frequency of consumption of each food multiplied by the amino acid content of the food for the appropriate portion size (31). All foods were classified as of either animal or vegetable origin and for mixed dishes the proportions contributed by animal and vegetable sources were calculated by breaking down the ingredients into foods that were attributable to a single source. Amino acid data are presented as a percentage of total energy intake in order to best represent the relative proportion to total dietary intake.

#### Assessment of covariates.

Intakes of energy and nutrients associated with arterial stiffness was determined from the FFQ as previously described. Height was measured to the nearest 0.5 cm with the use of a wall-mounted stadiometer, weight (light clothing only) was measured to the nearest 0.1 kg with digital scales, and BMI was calculated (kilograms per meter squared). Information on family medical history, medication use, lifestyle, and demographic variables were obtained by standardized nurse-administered questionnaire. Physical activity was classified as inactive, moderate, and active during work, home, and leisure time with the use of a questionnaire strongly correlated in this cohort with more in-depth assessment recording how much time subjects spent in moderate and vigorous nonweight-bearing and weight-bearing activity on average per week. The mean time spent in physical activity per week for each physical activity level was as follows: inactive, 16 min; light activity, 36 min; moderate activity, 102 min; and heavy activity, 199 min (32). Under-reporting of energy intake was based on a comparison of total predicted energy expenditure with reported energy intake and establishing quantitative limits to define under-reporters based on CIs calculated by taking into account within-subject variation in energy intake and expenditure (33, 34). Because excluding participants who under-report can introduce considerable bias, under-reporting was considered to be a covariate in all multivariable models (35).

#### Statistical analysis.

Statistical analyses were performed with Stata statistical software, version 11.2. The analysis focused on associations in twins as individuals, with SEs adjusted through robust regression with the use of the cluster option in Stata to take into account dependency within twin pairs. Quintiles of intake were calculated for total protein and the amino acids with known mechanistic links to outcomes associated with cardiovascular disease (arginine, cysteine, glutamic acid, glycine, histidine, leucine, and tyrosine). There was a significant interaction between amino acid intake and protein source for PWV, so all analyses were stratified by source (all, vegetable, and animal). ANCOVA was used to calculate adjusted means and evaluate statistical trends across these quintiles of intake. All models were adjusted for age (years); current smoking (yes or no); physical activity (inactive, moderately active, or active); BMI (kilograms per meter squared); use of hormone replacement therapy (yes or no); use of blood pressure or statin medication (yes or no); use of vitamin supplements (yes or no); use of oral contraceptives (yes or no); menopausal status (pre- or postmenopausal); family history of heart disease or hypertension (yes or no); under-reporting of dietary intake (yes or no); and intake of carbohydrates (percentage of energy); SFAs, MUFAs, and PUFAs (percentage of energy); alcohol (grams); and sodium, potassium, and magnesium (milligrams). In addition, models including individual amino acids were adjusted for intakes of the other 6 amino acids and PWV was adjusted for mean arterial pressure (MAP). Unadjusted values in the text are means ± SDs (IQRs) and adjusted values are means ± SEs. A *P* value < 0.05 was considered to be statistically significant.

## Results

The baseline characteristics of the population of 1898 female participants are presented in **Table 1**. A total of 83.3% (*n* = 1581) of participants classified as normotensive (pSBP <140 mm Hg and pDBP <90 mm Hg; data not shown). There were no differences in BMI, energy intake, or protein intake between the study participants and those excluded from the analyses; however, the participants were younger than those excluded (46.4 ± 12.4 y vs. 50.6 ± 12.9 y, *P* < 0.01; data not shown).

**TABLE 1:** Characteristics, vascular function, and dietary intake of the study population^1^

	Value
Characteristics	
Age, y	46.3 ± 12.4 (37.0, 56.0)
BMI, kg/m^2^	25.3 ± 4.5 (22.2, 27.4)
Female	100
Dizygotic	70.9
Current smoker	18.6
Physically active	22.7
Uses hormone replacement therapy	16.3
Uses blood pressure or statin medication	10.8
Uses vitamin supplements	54.7
Uses oral contraceptives	2.2
Postmenopausal	40.8
Has family history of hypertension or heart disease	38.2
Vascular function	
Peripheral systolic pressure, mm Hg	119 ± 16 (108, 129)
Peripheral diastolic pressure, mm Hg	76.2 ± 11.3 (68.0, 83.0)
Central systolic pressure, mm Hg	111 ± 17 (99, 121)
Central diastolic pressure, mm Hg	77.4 ± 11.5 (70.0, 84.8)
Central pulse pressure, mm Hg	33.5 ± 9.3 (27.0, 38.0)
Mean arterial pressure, mm Hg	92.3 ± 13.3 (76.4, 96.3)
Augmentation index, %	138 ± 26 (122, 156)
Pulse wave velocity,^2^ m/s	9.1 ± 1.8 (8.0, 10.1)
Intima–media thickness,^2^ mm	0.7 ± 0.2 (0.6, 0.8)
Dietary intake	
Energy, kcal/d	2007 ± 534 (1632, 2348)
Protein, g/d	80.2 ± 21.6 (65.3, 93.9)
Protein as a proportion of body weight, g/kg	1.2 ± 0.4 (1.0, 1.5)
Arginine, % protein/d	5.4 ± 0.4 (5.2, 5.7)
Cysteine, % protein/d	1.4 ± 0.1 (1.4, 1.5)
Glutamic acid, % protein/d	19.8 ± 1.1 (19.1, 20.4)
Glycine, % protein/d	4.0 ± 0.3 (3.8, 4.2)
Histidine, % protein/d	2.8 ± 0.1 (2.7, 2.9)
Leucine, % protein/d	7.9 ± 0.2 (7.8, 8.0)
Tyrosine, % protein/d	3.5 ± 0.1 (3.4, 3.5)
Carbohydrates, % energy/d	48.2 ± 6.3 (44.4, 52.2)
Saturated fat, % energy/d	11.6 ± 2.9 (9.65, 13.4)
Monounsaturated fat, % energy/d	10.4 ± 2.2 (9.0, 11.8)
Polyunsaturated fat, % energy/d	6.9 ± 1.7 (5.8, 8.0)
Alcohol, g/d	9.9 ± 13.6 (1.2, 13.4)
Sodium, mg/d	2300 ± 785 (1760, 2750)
Potassium, mg/d	4200 ± 1110 (3430, 4850)
Magnesium, mg/d	364 ± 100 (295, 422)

1 Values are means ± SDs (IQRs) or percentages, *n* = 1898.

2 Subset analysis for 728 participants.

**FIGURE 1:**
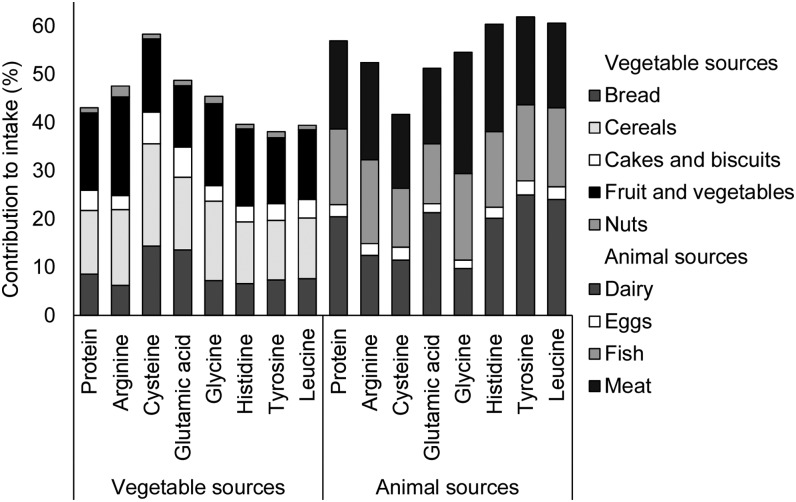
The percentage contribution of animal and vegetable foods to amino acid intake and key dietary sources in 1898 women aged 18–75 y.

Total protein intake was 85.1 ± 23.4 g/d and protein contributed 16.2% to total energy intake. Of the 7 amino acids investigated, glutamic acid (3.2% energy intake) and leucine (1.3% energy intake) made the greatest contribution to total energy intake. Animal and vegetable sources contributed a similar amount to intakes of arginine (52% animal), glutamic acid (51% animal), and glycine (55% animal), whereas intakes of histidine (60% animal), tyrosine (62% animal), and leucine (61% animal) was predominantly from animal sources (**Figure 1**). Conversely, vegetable sources contributed more to cysteine intake (42% animal). Generally, correlations between the amino acids investigated ranged from 0.03 (glutamic acid and leucine) to 0.61 (leucine and tyrosine), although stronger correlations were observed between glycine and leucine (0.70) and arginine and glycine (0.77).

In multivariable analyses, higher intakes of total protein and the 7 individual amino acids examined were significantly associated with lower peripheral and central blood pressure (**Table 2**), with the exception of total protein for central pulse pressure (cPP) (*P*-trend 0.06) and glycine for pDBP (*P*-trend 0.11) and central diastolic blood pressure (*P*-trend = 0.08). The magnitude of association was greatest for tyrosine intake, with differences between quintile 5 and quintile 1 of −5.6 ± 1.3 mm Hg for pSBP (*P*-trend < 0.01), −5.5 ± 1.3 mm Hg for central systolic blood pressure (cSBP) (*P*-trend < 0.01), −2.3 ± 0.7 mm Hg for cPP (*P*-trend < 0.01), and −4.0 ± 1.1 mm Hg for MAP (*P*-trend < 0.01) (Table 2). A higher leucine intake was also associated with significantly lower systolic blood pressure (peripheral and central), cPP, and MAP, with differences between extreme quintiles of intake of −5.4 ± 1.3 mm Hg (*P*-trend < 0.01), −5.5 ± 1.3 mm Hg (*P*-trend < 0.01), −2.6 ± 0.7 mm Hg (*P*-trend < 0.01), and −3.8 ± 1.1 mm Hg (*P*-trend < 0.01), respectively. For arterial stiffness, glutamic acid, leucine, and tyrosine intakes were significantly inversely associated with PWV (after adjustment for MAP) with respective differences of −0.4 ± 0.2 m/s (*P*-trend = 0.01), −0.4 ± 0.2 m/s (*P*-trend = 0.04) and −0.4 ± 0.2 m/s (*P*-trend = 0.02), comparing extremes of intake. Sensitivity analysis showed that the magnitude of associations with PWV were not markedly changed when protein was residually adjusted for energy intake (Q5–Q1, –0.2 ± 0.2 m/s, *P*-trend = 0.10), when amino acids were expressed as a percentage of protein (leucine Q5–Q1, –0.5 ± 0.2 m/s, *P*-trend 0.03; tyrosine Q5–Q1, –0.4 ± 0.2 m/s, *P*-trend = 0.04; glutamic acid Q5–Q1, –0.4 ± 0.2 m/s, *P*-trend = 0.19), or when potential under-reporters of energy intake were excluded from the analyses (leucine Q5–Q1, –0.6 ± 0.3 m/s, *P*-trend = 0.05; tyrosine Q5–Q1, –0.5 ± 0.3 m/s, *P*-trend = 0.06; glutamic acid Q5–Q1, –0.2 ± 0.3 m/s, *P*-trend = 0.08).

**TABLE 2:** Measures of central blood pressure and arterial stiffness by quintile of total protein and individual amino acids in women aged 18–75 y^1^

	Protein	Arginine	Cysteine	Glutamic acid	Glycine	Histidine	Leucine	Tyrosine
Intake, % energy								
Q1	12.8 ± 1.1	0.7 ± 0.07	0.1 ± 0.01	2.6 ± 0.1	0.5 ± 0.05	0.3 ± 0.03	1.0 ± 0.1	0.4 ± 0.04
Q2	14.8 ± 0.4	0.8 ± 0.02	0.2 ± 0.01	3.0 ± 0.1	0.6 ± 0.02	0.4 ± 0.01	1.2 ± 0.04	0.5 ± 0.02
Q3	16.1 ± 0.3	0.9 ± 0.02	0.2 ± 0.00	3.2 ± 0.1	0.6 ± 0.02	0.4 ± 0.01	1.3 ± 0.03	0.6 ± 0.01
Q4	17.4 ± 0.4	1.0 ± 0.03	0.2 ± 0.01	3.4 ± 0.1	0.7 ± 0.02	0.5 ± 0.01	1.4 ± 0.03	0.6 ± 0.02
Q5	19.9 ± 1.5	1.1 ± 0.1	0.3 ± 0.02	3.8 ± 0.3	0.8 ± 0.1	0.6 ± 0.05	1.6 ± 0.1	0.7 ± 0.1
pSBP, mm Hg								
Q1	120 ± 0.8	121 ± 0.9	120 ± 0.9	121 ± 0.9	121 ± 0.9	121 ± 0.9	122 ± 0.9	122 ± 0.9
Q2	121 ± 0.8	121 ± 0.8	122 ± 0.9	120 ± 0.8	120 ± 0.8	120 ± 0.8	121 ± 0.8	121 ± 0.8
Q3	119 ± 08	120 ± 0.8	120 ± 0.8	120 ± 0.8	121 ± 0.8	120 ± 0.8	120 ± 0.7	119 ± 0.8
Q4	119 ± 0.8	119 ± 0.8	118 ± 0.7	117 ± 0.8	118 ± 0.7	118 ± 0.8	118 ± 0.8	118 ± 0.8
Q5	118 ± 0.8	116 ± 0.9	118 ± 0.9	118 ± 0.9	117 ± 0.9	117 ± 0.9	116 ± 0.9	116 ± 0.9
* P*-trend	<0.01	<0.01	<0.01	<0.01	<0.01	<0.01	<0.01	<0.01
pDBP, mm Hg								
Q1	76.8 ± 0.5	76.8 ± 0.6	76.7 ± 0.6	77.4 ± 0.6	76.6 ± 0.6	77.0 ± 0.6	77.5 ± 0.6	77.7 ± 0.6
Q2	77.6 ± 0.5	76.9 ± 0.6	77.8 ± 0.6	77.1 ± 0.5	76.5 ± 0.6	76.9 ± 0.6	77.3 ± 0.5	77.3 ± 05
Q3	75.8 ± 0.6	76.6 ± 0.5	75.9 ± 0.6	76.0 ± 0.6	77.0 ± 0.6	76.7 ± 0.6	76.4 ± 0.5	76.3 ± 0.5
Q4	75.7 ± 0.5	75.6 ± 0.6	75.1 ± 0.5	74.8 ± 0.6	75.4 ± 0.5	75.3 ± 0.5	75.1 ± 0.5	75.1 ± 0.6
Q5	74.9 ± 0.6	74.8 ± 0.6	75.3 ± 0.6	75.5 ± 0.6	75.3 ± 0.7	74.9 ± 0.7	74.5 ± 0.6	74.5 ± 0.6
* P*-trend	<0.01	0.02	0.01	0.01	0.11	0.02	<0.01	<0.01
cSBP, mm Hg								
Q1	112 ± 0.8	112 ± 0.9	112 ± 0.8	113 ± 0.8	112 ± 0.9	113 ± 0.9	113 ± 0.8	113 ± 0.8
Q2	112 ± 0.8	112 ± 0.8	113 ± 0.8	112 ± 0.8	112 ± 0.8	112 ± 0.8	113 ± 0.8	112 ± 0.8
Q3	111 ± 0.8	112 ± 0.8	111 ± 0.8	111 ± 0.8	113 ± 0.8	112 ± 0.8	111 ± 0.7	111 ± 0.8
Q4	111 ± 0.8	110 ± 0.8	109 ± 0.7	109 ± 0.7	110 ± 0.7	110 ± 0.7	110 ± 0.8	110 ± 0.8
Q5	109 ± 0.8	108 ± 0.8	109 ± 0.8	109 ± 0.9	109 ± 0.9	109 ± 0.9	108 ± 0.8	108 ± 0.8
* P*-trend	<0.01	<0.01	<0.01	<0.01	0.01	<0.01	<0.01	<0.01
cDBP, mm Hg								
Q1	78.0 ± 0.6	78.1 ± 0.6	78.0 ± 0.6	78.7 ± 0.6	77.9 ± 0.6	78.3 ± 0.6	78.7 ± 0.6	78.9 ± 0.6
Q2	78.8 ± 0.6	78.1 ± 0.6	79.1 ± 0.6	78.3 ± 0.6	77.7 ± 0.6	78.1 ± 0.6	78.5 ± 0.6	78.5 ± 0.6
Q3	77.1 ± 0.6	78.0 ± 0.5	77.3 ± 0.6	77.2 ± 0.6	78.3 ± 0.6	77.9 ± 0.6	77.7 ± 0.6	77.4 ± 0.6
Q4	77.0 ± 0.6	76.9 ± 0.6	76.3 ± 0.5	76.1 ± 0.6	76.7 ± 0.6	76.7 ± 0.5	76.4 ± 0.6	76.5 ± 0.6
Q5	76.2 ± 0.6	75.9 ± 0.6	76.5 ± 0.7	76.8 ± 0.6	76.4 ± 0.7	76.1 ± 0.7	75.8 ± 0.6	75.7 v 0.6
* P*-trend	0.01	0.02	0.01	0.01	0.08	0.02	<0.01	<0.01
cPP, mm Hg								
Q1	34.1 ± 0.4	34.4 ± 0.5	33.9 ± 0.5	34.2 ± 0.5	34.0 ± 0.5	34.6 ± 0.5	34.5 ± 0.5	34.4 ± 0.5
Q2	33.4 ± 0.4	34.1 ± 0.4	34.1 ± 0.5	33.8 ± 0.4	33.8 ± 0.4	33.6 ± 0.4	34.0 ± 0.4	33.9 ± 0.4
Q3	33.7 ± 0.4	33.7 ± 0.4	34.0 ± 0.4	33.9 ± 0.5	34.3 ± 0.4	34.0 ± 0.4	33.4 ± 0.4	33.7 ± 0.4
Q4	33.7 ± 0.4	33.5 ± 0.4	32.9 ± 0.4	32.8 ± 0.4	32.9 ± 0.4	32.9 ± 0.4	33.6 ± 0.4	33.3 ± 0.4
Q5	32.6 ± 0.4	31.8 ± 0.5	32.6 ± 0.5	32.7 ± 0.5	32.5 ± 0.5	32.5 ± 0.5	31.9 ± 0.5	32.1 ± 0.5
* P*-trend	0.06	<0.01	0.02	0.02	0.02	0.01	<0.01	<0.01
MAP, mm Hg								
Q1	93.1 ± 0.6	93.1 ± 0.7	92.8 ± 0.7	93.7 ± 0.7	92.9 ± 0.7	93.4 ± 0.7	94.0 ± 0.7	94.1 ± 0.7
Q2	93.7 ± 0.6	93.5 ± 0.7	94.4 ± 0.7	93.4 ± 0.6	92.8 ± 0.7	93.3 ± 0.7	93.6 ± 0.6	93.8 ± 0.6
Q3	92.3 ± 0.6	93.0 ± 0.6	92.7 ± 0.7	92.3 ± 0.7	93.8 ± 0.6	93.2 ± 0.6	92.7 ± 0.6	92.4 ± 0.6
Q4	91.8 ± 0.6	92.0 ± 0.6	90.7 ± 0.6	90.7 ± 0.6	91.5 ± 0.6	91.4 ± 0.6	91.3 ± 0.6	91.3 ± 0.7
Q5	90.7 ± 0.7	90.0 ± 0.7	91.2 ± 0.7	91.7 ± 0.7	90.8 ± 0.8	90.5 ± 0.8	90.2 ± 0.7	90.1 ± 0.7
* P*-trend	<0.01	<0.01	<0.01	0.01	0.03	<0.01	<0.01	<0.01
PWV,^2^ m/s								
Q1	9.3 ± 0.1	9.1 ± 0.2	9.4 ± 0.2	9.3 ± 0.2	9.2 ± 0.2	9.2 ± 0.1	9.4 ± 0.2	9.4 ± 0.2
Q2	9.3 ± 0.1	9.2 ± 0.1	9.2 ± 0.1	9.5 ± 0.1	9.2 ± 0.1	9.4 ± 0.1	9.3 ± 0.1	9.3 ± 0.1
Q3	9.1 ± 0.1	9.2 ± 0.1	9.1 ± 0.1	9.0 ± 0.1	9.2 ± 0.1	9.1 ± 0.1	9.2 ± 0.1	9.2 ± 0.1
Q4	9.0 ± 0.1	9.1 ± 0.1	9.0 ± 0.1	9.0 ± 0.1	9.1 ± 0.1	9.0 ± 0.1	9.0 ± 0.1	8.9 ± 0.1
Q5	9.1 ± 0.1	9.1 ± 0.1	9.0 ± 0.1	8.9 ± 0.1	9.1 ± 0.1	9.0 ± 0.1	8.9 ± 0.1	9.0 ± 0.1
* P*-trend	0.06	0.79	0.05	0.01	0.69	0.15	0.02	0.02

1 Values are means ± SEs (except for Intake, for which values are unadjusted means ± SDs), *n* = 1898 (Q1 = 380, Q2 = 380, Q3 = 379, Q4 = 380, and Q5 = 379). Means are adjusted for age; current smoking; physical activity; BMI; use of hormone replacement therapy, blood pressure or statin medication, vitamin supplements, or oral contraceptives; menopausal status; family history of heart disease or hypertension; under-reporting of dietary intake; and intake of carbohydrates, SFAs, MUFAs, PUFAs, alcohol, sodium, potassium, and magnesium. In addition, individual amino acids were adjusted for intake of the other 6 amino acids, and PWV was adjusted for MAP. cDBP, central diastolic blood pressure; cPP, central pulse pressure; cSBP, central systolic blood pressure; MAP, mean arterial pressure; pDBP, peripheral diastolic blood pressure; pSBP, peripheral systolic blood pressure; PWV, pulse wave velocity; Q, quintile.

2 Subset analysis for 728 participants. Participant numbers were as follows: Q1 = 140, Q2 = 146, Q3 = 150, Q4 = 147, and Q5 = 145.

No significant associations were observed between intake of total protein or the individual amino acids assessed and either AI or IMT (data not shown). The results were not altered after exclusion of the low proportion (11%) of participants who reported taking blood pressure or statin medication (data not shown).

Higher intakes of amino acids from vegetable sources were inversely associated with cSBP and AI. Intakes of arginine, glycine, histidine, leucine and tyrosine were significantly associated with −2.0 to −2.4 mm Hg lower cSBP values and intake of cysteine, glutamic acid, lysine, and tyrosine with −3.5% to −5.7% lower AI, comparing extreme quintiles of intake (all *P*-trend < 0.05) (**Table 3**). Higher intakes of arginine and glycine from vegetable sources were also significantly associated with lower pSBP (arginine Q5–Q1, −2.3 ± 1.4 mm Hg, *P*-trend = 0.02; and glycine Q5–Q1, −2.6 ± 1.5 mm Hg, *P*-trend = 0.04) (Table 3). Furthermore, intake of arginine from vegetable sources was associated with lower cPP (Q5–Q1, −1.2 ± 0.8 mm Hg, *P*-trend = 0.04) and MAP (Q5–Q1, −1.2 ± 1.1 mm Hg, *P*-trend = 0.03). In relation to intakes of amino acids from animal sources, significant inverse associations were observed between glutamic acid, leucine, and tyrosine intakes and PWV, with respective differences of −0.3 ± 0.2 m/s (*P*-trend = 0.03), −0.3 ± 0.2 m/s (*P*-trend = 0.02), and −0.4 ± 0.2 m/s (*P*-trend = 0.02) between extreme quintiles of intake. Total vegetable protein intake and total animal protein intake were not associated with any of the outcomes assessed (*P*-trend all > 0.05), and there were no significant associations for IMT (data not shown).

**TABLE 3:** Measures of central blood pressure and arterial stiffness between extreme quintiles of total protein and individual amino acids from vegetable and animal sources in women aged 18–75 y^1^

	Protein	Arginine	Cysteine	Glutamic acid	Glycine	Histidine	Lysine	Tyrosine
Vegetable sources	
Intake, % energy	
Q1	4.6 ± 0.4	0.3 ± 0.03	0.1 ± 0.01	1.0 ± 0.1	0.2 ± 0.02	0.1 ± 0.01	0.3 ± 0.04	0.1 ± 0.02
Q2	5.4 ± 0.2	0.3 ± 0.01	0.1 ± 0.00	1.2 ± 0.05	0.2 ± 0.01	0.1 ± 0.00	0.4 ± 0.03	0.2 ± 0.01
Q3	6.0 ± 0.2	0.3 ± 0.01	0.1 ± 0.00	1.3 ± 0.04	0.3 ± 0.01	0.2 ± 0.00	0.4 ± 0.03	0.2 ± 0.02
Q4	6.6 ± 0.2	0.4 ± 0.01	0.1 ± 0.00	1.5 ± 0.06	0.3 ± 0.01	0.2 ± 0.01	0.5 ± 0.03	0.2 ± 0.02
Q5	8.0 ± 0.9	0.5 ± 0.1	0.2 ± 0.02	1.8 ± 0.2	0.3 ± 0.04	0.2 ± 0.03	0.6 ± 0.1	0.2 ± 0.04
pSBP, mm Hg	
Q5–Q1	−2.6 ± 1.5	−2.3 ± 1.4	0.3 ± 1.7	−0.2 ± 1.7	−2.6 ± 1.5	−2.1 ± 1.4	−2.0 ± 1.4	−2.0 ± 1.4
* P*-trend	0.12	0.02	0.96	0.78	0.04	0.05	0.05	0.05
pDBP, mm Hg	
Q5–Q1	−1.9 ± 1.0	−0.9 ± 0.9	−1.0 ± 1.1	−1.0 ± 1.1	−1.1 ± 1.0	−1.6 ± 1.0	−1.6 ± 0.9	−1.6 ± 0.9
* P*-trend	0.07	0.08	0.29	0.27	0.15	0.05	0.03	0.04
cSBP, mm Hg	
Q5–Q1	−2.8 ± 1.5	−2.1 ± 1.3	−0.2 ± 1.6	−0.7 ± 1.6	−2.4 ± 1.4	−2.1 ± 1.4	−2.0 ± 1.3	−2.0 ± 1.3
* P*-trend	0.08	0.02	0.71	0.63	0.03	0.04	0.03	0.04
cDBP, mm Hg	
Q5–Q1	−1.7 ± 1.0	−1.0 ± 1.0	−0.8 ± 1.1	−0.7 ± 1.1	−1.0 ± 1.0	−1.5 ± 1.0	−1.3 ± 1.0	−1.3 ± 1.0
* * *P*-trend	0.11	0.09	0.39	0.41	0.17	0.07	0.07	0.07
cPP, mm Hg	
Q5–Q1	−1.2 ± 0.9	−1.2 ± 0.8	0.5 ± 0.9	−0.1 ± 0.9	−1.4 ± 0.8	−0.6 ± 0.8	−0.7 ± 0.7	−0.7 ± 0.7
* P*-trend	0.29	0.04	0.66	0.88	0.04	0.23	0.15	0.15
MAP, %	
Q5–Q1	−1.8 ± 1.2	−1.2 ± 1.1	−0.6 ± 1.3	−0.2 ± 1.3	−1.7 ± 1.2	−1.8 ± 1.1	−1.5 ± 1.1	−1.5 ± 1.1
* P*-trend	0.13	0.03	0.51	0.71	0.05	0.05	0.06	0.06
AI, mm Hg	
Q5–Q1	−3.8 ± 2.3	−1.6 ± 2.2	−4.3 ± 2.3	−5.7 ± 2.4	−1.7 ± 2.2	−1.6 ± 2.3	−3.5 ± 2.1	−3.6 ± 2.1
* P*-trend	0.07	0.18	0.03	0.03	0.06	0.19	0.03	0.03
PWV,^2^ mm Hg	
Q5–Q1	−0.2 ± 0.2	0.1 ± 0.2	−0.2 ± 0.2	−0.3 ± 0.2	−0.0 ± 0.3	−0.1 ± 0.2	−0.2 ± 0.2	−0.2 ± 0.2
* P*-trend	0.32	0.74	0.15	0.21	0.99	0.65	0.26	0.46
Animal sources	
Intake, % energy	
Q1	6.3 ± 1.2	0.3 ± 0.1	0.1 ± 0.01	1.2 ± 0.2	0.2 ± 0.05	0.2 ± 0.04	0.5 ± 0.1	0.2 ± 0.04
Q2	8.6 ± 0.4	0.4 ± 0.02	0.1 ± 0.05	1.6 ± 0.1	0.3 ± 0.02	0.3 ± 0.01	0.7 ± 0.04	0.3 ± 0.02
Q3	9.9 ± 0.4	0.5 ± 0.02	0.1 ± 0.02	1.8 ± 0.1	0.4 ± 0.02	0.3 ± 0.01	0.8 ± 0.04	0.4 ± 0.02
Q4	11.4 ± 0.5	0.6 ± 0.03	0.1 ± 0.01	2.1 ± 0.1	0.4 ± 0.02	0.3 ± 0.02	0.9 ± 0.05	0.4 ± 0.02
Q5	14.1 ± 1.6	0.8 ± 0.1	0.2 ± 0.02	2.5 ± 0.3	0.6 ± 0.1	0.4 ± 0.05	1.2 ± 0.1	0.5 ± 0.1
pSBP, mm Hg	
Q5–Q1	−1.9 ± 1.4	−2.2 ± 1.3	−1.3 ± 1.3	−2.2 ± 1.3	−1.8 ± 1.3	−2.1 ± 1.4	−2.1 ± 1.3	−2.1 ± 1.3
* P*-trend	0.17	0.07	0.30	0.21	0.12	0.17	0.11	0.1
pDBP, mm Hg	
Q5–Q1	−1.6 ± 1.0	−1.7 ± 0.9	−1.4 ± 1.0	−2.0 ± 1.0	−1.4 ± 1.0	−1.4 ± 1.0	−1.9 ± 1.0	−1.9 ± 1.0
* P*-trend	0.11	0.10	0.19	0.07	0.14	0.12	0.05	0.05
cSBP, mm Hg	
Q5–Q1	−2.1 ± 1.3	−2.2 ± 1.3	−1.5 ± 1.3	−2.5 ± 1.3	−1.7 ± 1.3	−2.2 ± 1.4	−2.3 ± 1.3	−2.4 ± 1.3
* P*-trend	0.15	0.10	0.30	0.17	0.16	0.16	0.10	0.08
cDBP, mm Hg	
Q5–Q1	−1.5 ± 1.0	−1.7 ± 0.9	−1.5 ± 1.0	−1.9 ± 1.0	−1.4 ± 1.0	−1.3 ± 1.0	−1.7 ± 1.0	−1.8 ± 1.0
* P*-trend	0.18	0.13	0.22	0.11	0.15	0.18	0.09	0.08
cPP, mm Hg	
Q5–Q1	−0.6 ± 0.7	−0.5 ± 0.7	−0.0 ± 0.7	−0.6 ± 0.7	−0.3 ± 0.7	−0.9 ± 0.7	−0.6 ± 0.7	−0.6 ± 0.7
* P*-trend	0.43	0.35	0.84	0.73	0.54	0.44	0.50	0.47
MAP, %	
Q5–Q1	−1.4 ± 1.1	−1.7 ± 1.1	−1.3 ± 1.1	−1.7 ± 1.1	−1.5 ± 1.1	−1.5 ± 1.1	−1.6 ± 1.1	−1.7 ± 1.1
* P*-trend	0.29	0.16	0.36	0.25	0.19	0.23	0.17	0.14
AI, mm Hg	
Q5–Q1	−1.4 ± 2.2	−0.1 ± 2.1	−1.3 ± 2.1	−1.5 ± 2.2	−0.9 ± 2.2	−0.9 ± 2.2	−2.1 ± 2.2	−2.5 ± 2.1
* P*-trend	0.85	0.71	0.79	0.75	0.85	0.95	0.73	0.58
PWV,^2^ mm Hg	
Q5–Q1	−0.1 ± 0.2	−0.1 ± 0.2	−0.2 ± 0.2	−0.3 ± 0.2	−0.0 ± 0.2	−0.1 ± 0.2	−0.3 ± 0.2	−0.4 ± 0.2
* P*-trend	0.11	0.44	0.25	0.03	0.69	0.22	0.02	0.02

1 Values are means ± SEs between quintile 5 and quintile 1 (except for Intake, for which values are unadjusted means ± SDs), *n* = 1898 (Q1 = 380, Q2 = 380, Q3 = 379, Q4 = 380, Q5 = 379). Means are adjusted for age; current smoking; physical activity; BMI; use of hormone replacement therapy, blood pressure or statin medication, vitamin supplements, or oral contraceptives; menopausal status; family history of heart disease or hypertension; under-reporting of dietary intake; and intakes of carbohydrates, SFAs, MUFAs, PUFAs, alcohol, sodium, potassium, and magnesium. Intakes of total protein and individual amino acids from animal sources and intakes from vegetable sources were mutually adjusted for one another, and in addition PWV was adjusted for MAP. AI, augmentation index; cDBP, central diastolic blood pressure; cPP, central pulse pressure; cSBP, central systolic blood pressure; MAP, mean arterial pressure; pDBP, peripheral diastolic blood pressure; pSBP, peripheral systolic blood pressure; PWV, pulse wave velocity; Q, quintile.

2 Subset analysis for 728 participants. Participant numbers were as follows: Q1 = 140, Q2 = 146, Q3 = 150, Q4 = 147, and Q5 = 145.

## Discussion

To our knowledge, this is the first cross-sectional study to examine associations between amino acids that have known mechanistic links to cardiovascular disease and a range of in vivo measures of arterial stiffness and central blood pressure associated with cardiovascular disease risk. We showed that higher intakes of all 7 of the amino acids examined (arginine, cysteine, glutamic acid, glycine, histidine, leucine, and tyrosine) were significantly associated with improved measures of peripheral and central blood pressure. Higher intakes of glutamic acid, leucine, and tyrosine were also associated with lower PWV. These associations were significant after adjustment for a number of important covariates known to be associated with vascular health, including lifestyle factors, medication use, and other nutrients. The magnitude of the inverse associations ranged from −2.8 to −5.5 mm Hg for systolic blood pressure, −1.5 to −3.2 mm Hg for central diastolic blood pressure, and −0.40 to −0.45 m/s for PWV.

It was previously estimated that a modest reduction in systolic blood pressure of 5 mm Hg would potentially lead to an overall reduction in mortality from stroke, coronary heart disease, or all-cause mortality (36). Intakes of the amino acids investigated in the current study were associated with a mean difference in pSBP of −4.1 mm Hg (range: −2.6 to −5.6 mm Hg). The magnitude of these associations is similar to those previously reported for established lifestyle risk factors for hypertension, including sodium intake, physical activity, and alcohol consumption (37). For PWV, the scale of the association was 0.4 m/s, which is similar to the magnitude of change previously associated with not smoking supplementation with n–3 FAs and, to the differences observed between individuals with or without metabolic syndrome, hypertension, or hypercholesterolemia (38, 39).

Our results provide further insights into the vascular effects of these amino acids and a potential explanation to support the reduction in blood pressure observed with higher protein intake in a recent meta-analysis (2). Our findings are consistent with randomized, controlled trials reporting decreases in pSBP of 2 mm Hg with 40 g of milk or soy protein and 1.4 mm Hg with a diet substituting 10% of energy from carbohydrate with that from protein (40, 41). Furthermore, cross-sectional studies of amino acid intake and blood pressure have reported intakes of tyrosine and glutamic acid to be associated with, on average, a 2 mm Hg reduction in pSBP (14, 15), and intake of histidine with a 4% reduced risk of an increase in pSBP of 16 mm Hg (16).

These current results, however, do not support the findings of the INTERMAP (INTERnational study of MAcro- and micronutrients and blood Pressure), which showed that higher glycine intake was associated with an increase in blood pressure, and those of the Rotterdam study, which found no significant associations between intakes of glutamic acid, arginine, and cysteine and blood pressure or risk of hypertension (14, 17). A number of factors, including study design, could account for these divergent findings; interestingly, there were differences in habitual dietary patterns between studies and consequently in the major sources of amino acids. For example, in the INTERMAP, meat contributed to 33–47% of dietary glycine intake and fish to 6–13%, compared with 25% and 18%, respectively, in our participants. Food sources of protein vary greatly in their nonprotein constituents and have previously been shown to have differing associations with cardiovascular disease risk in women, with red meat associated with a 13% increased risk and fish intake with a 19% reduced risk (42).

Because of the strong interrelations previously reported between blood pressure and protein source, and because we observed a significant interaction in the current analyses, we further examined the relation between amino acid intake and arterial stiffness according to protein source. Protein from vegetable sources, but not animal sources, has previously been shown to be associated with a −1.1 mm Hg reduction in pSBP and 15% reduction in risk of hypertension (3, 4). We found that higher intakes of amino acids from vegetable sources, but not animal sources, were associated with lower systolic blood pressure, MAP, and AI. Intakes of glutamic acid, leucine, and tyrosine from animal sources were associated with lower a PWV of 0.3–0.4 m/s between extreme quintiles of intake. These data provide support for a previous study from Japan reporting an inverse relation between higher animal protein intake and blood pressure (43), but do not support those studies in Western populations in which animal protein was found to be unrelated to blood pressure (3). Fish in the current study contributed >3 times more to glutamic acid intake than previously reported in a UK cohort, which may offer some explanation for this finding (15). Studies in hypertensive rats provide evidence to suggest that protein from fish is more effective at lowering blood pressure than casein over a 2 mo period (44), and a meta-analysis of adult human trials revealed that n–3 FAs were effective at improving PWV by 0.33 m/s (39). Fish intake has also been associated with overall diet quality (5). The underlying mechanism of interaction between amino acid intake and PWV observed in the current study is unclear, and it must be considered that in our study animal sources contributed more to leucine and tyrosine than vegetable sources; these analyses may be reflecting our findings for intake from all sources, which were inherently adjusted for protein source. The reasons why we observed associations with total protein and not animal protein when the correlation between total and animal protein intakes was so high (*r* = 0.89, *P* < 0.01) remains unclear.

The findings of the current study reflect intakes of amino acids that are readily achievable in the habitual diet. The difference in PWV of 0.4 m/s shown between extreme quintiles of intakes of glutamic acid, leucine, and tyrosine equates to a 3.53 g, 1.64 g, and 0.76 g difference in intake, respectively. This intake can be incorporated into the diet by consuming approximately one-half of a medium steak (74 g), a medium salmon fillet (100 g), or a 500 mL glass of skimmed milk.

Strengths of the current study include the large sample of well-characterized participants and the rigorous assessment of a range of in vivo measures of arterial stiffness and central blood pressure. It is well established that aortic stiffness, as assessed by PWV, is able to predict future cardiovascular events and all-cause mortality (45). The publication of reference values for PWV and the addition of PWV to traditional risk biomarkers in recent guidelines for hypertension management demonstrate prognostic ability (46, 47). It was notable that the associations observed in the current analysis were shown in a population in which over 80% were classified as normotensive, and it is plausible that any associations would be more pronounced in a hypertensive group. These participants previously have been shown to be representative of the general population in terms of blood pressure and diet (23, 24), and these data have been used in previous studies of dietary exposure (48). The FFQ used in the current study was used to rank participants according to their amino acid intake. Although the FFQ has not been shown to accurately quantify amino acid intake, it has previously been validated against 24 h recalls and shown to classify over 85% of participants into the same or adjacent quintile of protein intake, demonstrating its ability to rank participants according to their habitual protein intake (49).

The limitations of the current study include the cross-sectional design that meant we were unable to infer causality Numerous hypothesis-driven comparisons were made in our analysis that we felt were justified, given the novel and exploratory nature of the analyses. The inclusion of twin pairs within the same sample may introduce bias, but this was reduced by accounting for the clustering within twin pairs in all statistical analyses and with the use of a sample shown to be representative of the general population in terms of outcome variables and dietary intake. Validated biomarkers, such as 24 h urine nitrogen, are available for total protein intake, but they were not measured in the current study and may have reduced potential measurement error (50). Furthermore, residual confounding may have occurred despite the multivariable modeling adjusted for a range of dietary and lifestyle confounder variables (such as age, smoking, physical activity, BMI, medication use, and intakes of other nutrients associated with vascular health).

In conclusion, these novel data suggest that intakes of selected amino acids is associated with arterial stiffness and central blood pressure, with significant associations observed for PWV and cSBP similar in magnitude to established lifestyle risk factors for hypertension, such as physical activity, not smoking, and reduced intakes of sodium and alcohol (37, 38). The intakes of amino acids associated with lower arterial stiffness and central blood pressure are easily achievable in the habitual diet, making these findings very relevant for public health strategies to reduce cardiovascular disease risk. Our findings highlight the need for more intervention trials examining dietary achievable intakes of amino acids and cardiovascular outcomes.

## Footnotes

5 Abbreviations used: AI, augmentation index; cPP, central pulse pressure; cSBP, central systolic blood pressure; IMT, intima–media thickness; INTERMAP, INTERnational study of MAcro- and micronutrients and blood Pressure; MAP, mean arterial pressure; pDBP, peripheral diastolic blood pressure; pSBP, peripheral systolic blood pressure; PWV, pulse wave velocity.

## References

[1] Stamler J, Elliott P, Kesteloot H, Nichols R, Claeys G, Dyer AR, Stamler R Inverse relation of dietary protein markers with blood pressure. Findings for 10,020 men and women in the INTERSALT Study. INTERSALT Cooperative Research Group. INTERnational study of SALT and blood pressure. Circulation 1996;94:1629–34. 8840854 10.1161/01.cir.94.7.1629

[2] Rebholz CM, Friedman EE, Powers LJ, Arroyave WD, He J, Kelly TN Dietary protein intake and blood pressure: a meta-analysis of randomized controlled trials. Am J Epidemiol 2012;176 Suppl 7:S27–43. 23035142 10.1093/aje/kws245

[3] Elliott P, Stamler J, Dyer AR, Appel L, Dennis B, Kesteloot H, Ueshima H, Okayama A, Chan Q, Garside DB, Association between protein intake and blood pressure: the INTERMAP Study. Arch Intern Med 2006;166:79–87. 16401814 10.1001/archinte.166.1.79 PMC6593153

[4] Altorf-van der Kuil W, Engberink MF, Vedder MM, Boer JM, Verschuren WM, Geleijnse JM Sources of dietary protein in relation to blood pressure in a general Dutch population. PLoS One 2012;7:e30582. 22347387 10.1371/journal.pone.0030582 PMC3274530

[5] Camilleri GM, Verger EO, Huneau JF, Carpentier F, Dubuisson C, Mariotti F Plant and animal protein intakes are differently associated with nutrient adequacy of the diet of French adults. J Nutr 2013;143:1466–73. 23864509 10.3945/jn.113.177113

[6] Prasad A, Andrews NP, Padder FA, Husain M, Quyyumi AA Glutathione reverses endothelial dysfunction and improves nitric oxide bioavailability. J Am Coll Cardiol 1999;34:507–14. 10440166 10.1016/s0735-1097(99)00216-8

[7] El Hafidi M, Perez I, Banos G Is glycine effective against elevated blood pressure? Curr Opin Clin Nutr Metab Care 2006;9:26–31. 16444815 10.1097/01.mco.0000196143.72985.9a

[8] Vasdev S, Singal P, Gill V The antihypertensive effect of cysteine. Int J Angiol 2009;18:7–21. 22477470 10.1055/s-0031-1278316 PMC2721729

[9] Toba H, Nakamori A, Tanaka Y, Yukiya R, Tatsuoka K, Narutaki M, Tokitaka M, Hariu H, Kobara M, Nakata T Oral L-histidine exerts antihypertensive effects via central histamine H3 receptors and decreases nitric oxide content in the rostral ventrolateral medulla in spontaneously hypertensive rats. Clin Exp Pharmacol Physiol 2010;37:62–8. 19566844 10.1111/j.1440-1681.2009.05227.x

[10] Baum JI, O’Connor JC, Seyler JE, Anthony TG, Freund GG, Layman DK Leucine reduces the duration of insulin-induced PI 3-kinase activity in rat skeletal muscle. Am J Physiol Endocrinol Metab 2005;288:E86–91. 15339747 10.1152/ajpendo.00272.2004

[11] Gazit V, Ben-Abraham R, Vofsi O, Katz Y L-cysteine increases glucose uptake in mouse soleus muscle and SH-SY5Y cells. Metab Brain Dis 2003;18:221–31. 14567472 10.1023/a:1025507216746

[12] Blouet C, Mariotti F, Azzout-Marniche D, Mathe V, Mikogami T, Tome D, Huneau JF Dietary cysteine alleviates sucrose-induced oxidative stress and insulin resistance. Free Radic Biol Med 2007;42:1089–97. 17349935 10.1016/j.freeradbiomed.2007.01.006

[13] Fernstrom JD, Fernstrom MH Tyrosine, phenylalanine, and catecholamine synthesis and function in the brain. J Nutr 2007;137:1539S–1547S; discussion 1548S. 17513421 10.1093/jn/137.6.1539S

[14] Altorf-van der Kuil W, Engberink MF, De Neve M, van Rooij FJ, Hofman A, van’t Veer P, Witteman JC, Franco OH, Geleijnse JM Dietary amino acids and the risk of hypertension in a Dutch older population: the Rotterdam Study. Am J Clin Nutr 2013;97:403–10. 23283504 10.3945/ajcn.112.038737

[15] Stamler J, Brown IJ, Daviglus ML, Chan Q, Kesteloot H, Ueshima H, Zhao L, Elliott P, Group IR Glutamic acid, the main dietary amino acid, and blood pressure: the INTERMAP Study (International Collaborative Study of Macronutrients, Micronutrients and Blood Pressure). Circulation 2009;120:221–8. 19581495 10.1161/CIRCULATIONAHA.108.839241 PMC4048930

[16] Tuttle KR, Milton JE, Packard DP, Shuler LA, Short RA Dietary amino acids and blood pressure: A cohort study of patients with cardiovascular disease. Am J Kidney Dis 2012;59:803–9. 22381643 10.1053/j.ajkd.2011.12.026

[17] Stamler J, Brown IJ, Daviglus ML, Chan Q, Miura K, Okuda N, Ueshima H, Zhao L, Elliott P Dietary glycine and blood pressure: The international study on macro/micronutrients and blood pressure. Am J Clin Nutr 2013;98:136–45. 23656904 10.3945/ajcn.112.043000 PMC3683815

[18] Nagata C, Wada K, Tamura T, Kawachi T, Konishi K, Tsuji M, Nakamura K Dietary intakes of glutamic acid and glycine are associated with stroke mortality in Japanese adults. J Nutr 2015;145:720–8. 25833775 10.3945/jn.114.201293

[19] Larsson SC, Hakansson N, Wolk A Dietary cysteine and other amino acids and stroke incidence in women. Stroke 2015;46:922–6. 25669310 10.1161/STROKEAHA.114.008022

[20] Laurent S, Cockcroft J, Van Bortel L, Boutouyrie P, Giannattasio C, Hayoz D, Pannier B, Vlachopoulos C, Wilkinson I, Struijker-Boudier H, Expert consensus document on arterial stiffness: methodological issues and clinical applications. Eur Heart J 2006;27:2588–605. 17000623 10.1093/eurheartj/ehl254

[21] Chambless LE, Heiss G, Folsom AR, Rosamond W, Szklo M, Sharrett AR, Clegg LX Association of coronary heart disease incidence with carotid arterial wall thickness and major risk factors: The Atherosclerosis Risk in Communities (ARIC) Study, 1987–1993. Am J Epidemiol 1997;146:483–94. 9290509 10.1093/oxfordjournals.aje.a009302

[22] Andrew T, Hart DJ, Snieder H, de Lange M, Spector TD, MacGregor AJ Are twins and singletons comparable? A study of disease-related and lifestyle characteristics in adult women. Twin Res 2001;4:464–77. 11780939 10.1375/1369052012803

[23] Teucher B, Skinner J, Skidmore PM, Cassidy A, Fairweather-Tait SJ, Hooper L, Roe MA, Foxall R, Oyston SL, Cherkas LF, Dietary patterns and heritability of food choice in a UK female twin cohort. Twin Res Hum Genet 2007;10:734–48. 17903115 10.1375/twin.10.5.734

[24] Cecelja M, Jiang B, McNeill K, Kato B, Ritter J, Spector T, Chowienczyk P Increased wave reflection rather than central arterial stiffness is the main determinant of raised pulse pressure in women and relates to mismatch in arterial dimensions: a twin study. J Am Coll Cardiol 2009;54:695–703. 19679247 10.1016/j.jacc.2009.04.068

[25] Snieder H, Hayward CS, Perks U, Kelly RP, Kelly PJ, Spector TD Heritability of central systolic pressure augmentation: a twin study. Hypertension 2000;35:574–9. 10679500 10.1161/01.hyp.35.2.574

[26] Cecelja M, Jiang B, Bevan L, Frost ML, Spector TD, Chowienczyk PJ Arterial stiffening relates to arterial calcification but not to noncalcified atheroma in women. A twin study. J Am Coll Cardiol 2011;57:1480–6. 21435518 10.1016/j.jacc.2010.09.079 PMC3919172

[27] Bingham SA, Gill C, Welch A, Cassidy A, Runswick SA, Oakes S, Lubin R, Thurnham DI, Key TJ, Roe L, Validation of dietary assessment methods in the UK arm of EPIC using weighed records, and 24-hour urinary nitrogen and potassium and serum vitamin C and carotenoids as biomarkers. Int J Epidemiol 1997;26 Suppl 1:S137–51. 9126542 10.1093/ije/26.suppl_1.s137

[28] Bingham SA, Welch AA, McTaggart A, Mulligan AA, Runswick SA, Luben R, Oakes S, Khaw KT, Wareham N, Day NE Nutritional methods in the European Prospective Investigation of Cancer in Norfolk. Public Health Nutr 2001;4:847–58. 11415493 10.1079/phn2000102

[29] Paul AA SD, Russell J. First supplement to McCance and Widdowsen’s the composition of foods. London: HMSO, 1980.

[30] US Department of Agriculture. USDA national nutrient database for standard reference: release 24. Washington (DC): 2011.

[31] Crawley H Food portion sizes. London: H.M. Stationary Office 2002.

[32] Cherkas LF, Hunkin JL, Kato BS, Richards JB, Gardner JP, Surdulescu GL, Kimura M, Lu X, Spector TD, Aviv A The association between physical activity in leisure time and leukocyte telomere length. Arch Intern Med 2008;168:154–8. 18227361 10.1001/archinternmed.2007.39

[33] Institute of Medicine of the National Academies. Dietary Reference Intakes for energy, carbohydrate, fiber, fat, fatty acids, cholesterol, protein and amino acids. Washington (DC): National Academies Press, 2002. 10.1016/s0002-8223(02)90346-9 12449285

[34] Black AE, Cole TJ Within- and between-subject variation in energy expenditure measured by the doubly-labelled water technique: implications for validating reported dietary energy intake. Eur J Clin Nutr 2000;54:386–94. 10822285 10.1038/sj.ejcn.1600970

[35] Rennie KL, Coward A, Jebb SA Estimating under-reporting of energy intake in dietary surveys using an individualised method. Br J Nutr 2007;97:1169–76. 17433123 10.1017/S0007114507433086

[36] Stamler R Implications of the INTERSALT study. Hypertension 1991;17:I16–20. 1986996 10.1161/01.hyp.17.1_suppl.i16

[37] Whelton PK, He J, Appel LJ, Cutler JA, Havas S, Kotchen TA, Roccella EJ, Stout R, Vallbona C, Winston MC, Primary prevention of hypertension: clinical and public health advisory from The National High Blood Pressure Education Program. JAMA 2002;288:1882–8. 12377087 10.1001/jama.288.15.1882

[38] McEniery CM, Yasmin, Maki-Petaja KM, McDonnell BJ, Munnery M, Hickson SS, Franklin SS, Cockcroft JR, Wilkinson IB, Anglo-Cardiff Collaboration Trial Investigators. The impact of cardiovascular risk factors on aortic stiffness and wave reflections depends on age: the Anglo-Cardiff Collaborative Trial (ACCT III). Hypertension 2010;56:591–7. 20696989 10.1161/HYPERTENSIONAHA.110.156950

[39] Pase MP, Grima NA, Sarris J Do long-chain n-3 fatty acids reduce arterial stiffness? A meta-analysis of randomised controlled trials. Br J Nutr 2011;106:974–80. 22005318 10.1017/S0007114511002819

[40] He J, Wofford MR, Reynolds K, Chen J, Chen CS, Myers L, Minor DL, Elmer PJ, Jones DW, Whelton PK Effect of dietary protein supplementation on blood pressure: a randomized, controlled trial. Circulation 2011;124:589–95. 21768541 10.1161/CIRCULATIONAHA.110.009159 PMC3150301

[41] Appel LJ, Sacks FM, Carey VJ, Obarzanek E, Swain JF, Miller ER 3rd, Conlin PR, Erlinger TP, Rosner BA, Laranjo NM, Effects of protein, monounsaturated fat, and carbohydrate intake on blood pressure and serum lipids: results of the OmniHeart randomized trial. JAMA 2005;294:2455–64. 16287956 10.1001/jama.294.19.2455

[42] Bernstein AM, Sun Q, Hu FB, Stampfer MJ, Manson JE, Willett WC Major dietary protein sources and risk of coronary heart disease in women. Circulation 2010;122:876–83. 20713902 10.1161/CIRCULATIONAHA.109.915165 PMC2946797

[43] Umesawa M, Sato S, Imano H, Kitamura A, Shimamoto T, Yamagishi K, Tanigawa T, Iso H Relations between protein intake and blood pressure in Japanese men and women: the Circulatory Risk in Communities Study (CIRCS). Am J Clin Nutr 2009;90:377–84. 19515740 10.3945/ajcn.2008.27109

[44] Ait-Yahia D, Madani S, Savelli JL, Prost J, Bouchenak M, Belleville J Dietary fish protein lowers blood pressure and alters tissue polyunsaturated fatty acid composition in spontaneously hypertensive rats. Nutrition 2003;19:342–6. 12679169 10.1016/s0899-9007(02)00858-4

[45] Vlachopoulos C, Aznaouridis K, Stefanadis C Prediction of cardiovascular events and all-cause mortality with arterial stiffness: a systematic review and meta-analysis. J Am Coll Cardiol 2010;55:1318–27. 20338492 10.1016/j.jacc.2009.10.061

[46] Mancia G, Fagard R, Narkiewicz K, Redon J, Zanchetti A, Bohm M, Christiaens T, Cifkova R, De Backer G, Dominiczak A, 2013 ESH/ESC guidelines for the management of arterial hypertension: the Task Force for the Management of Arterial Hypertension of the European Society of Hypertension (ESH) and of the European Society of Cardiology (ESC). Eur Heart J 2013;34:2159–219. 23771844 10.1093/eurheartj/eht151

[47] Reference Values for Arterial Stiffness Collaboration. Determinants of pulse wave velocity in healthy people and in the presence of cardiovascular risk factors: ‘establishing normal and reference values.’ Eur Heart J 2010;31:2338–50. 20530030 10.1093/eurheartj/ehq165 PMC2948201

[48] Jennings A, Welch AA, Fairweather-Tait SJ, Kay C, Minihane AM, Chowienczyk P, Jiang BY, Cecelja M, Spector T, Macgregor A, Higher anthocyanin intake is associated with lower arterial stiffness and central blood pressure in women. Am J Clin Nutr 2012;96:781–8. 22914551 10.3945/ajcn.112.042036

[49] Kroke A, Klipstein-Grobusch K, Voss S, Moseneder J, Thielecke F, Noack R, Boeing H Validation of a self-administered food-frequency questionnaire administered in the European Prospective Investigation into Cancer and Nutrition (EPIC) Study: comparison of energy, protein, and macronutuient intakes estimated with the doubly labeled water, urinary nitrogen, and repeated 24-h dietary recall methods. Am J Clin Nutr 1999;70:439–47. 10500011 10.1093/ajcn/70.4.439

[50] Bingham SA Urine nitrogen as a biomarker for the validation of dietary protein intake. J Nutr 2003;133 Suppl 3:921S–4S. 12612177 10.1093/jn/133.3.921S

